# Leveraging a Genetic Proxy to Investigate the Effects of Lifelong Cardiac Sodium Channel Blockade

**DOI:** 10.1161/CIRCULATIONAHA.125.075057

**Published:** 2025-11-24

**Authors:** Julian S. Wanner, Maren Krafft, Teemu Niiranen, Dominic S. Zimmerman, FinnGen, Patrick T. Ellinor, Girish Nadkarni, Sean J. Jurgens, Joel Rämö, Henrike O. Heyne

**Affiliations:** Hasso Plattner Institute, Digital Engineering Faculty, University of Potsdam, Germany (J.S.W., M.K., H.O.H.).; Institute for Molecular Medicine Finland (FIMM), HiLIFE, University of Helsinki, Finland (J.S.W., J.R., H.O.H.).; Population Health Unit, Finnish Institute for Health and Welfare, Helsinki, Finland (T.N.).; Cardiovascular Disease Initiative, Broad Institute of MIT and Harvard, Cambridge, MA (P.T.E., S.J.J., J.R.).; Hasso Plattner Institute for Digital Health at Mount Sinai, Mount Sinai School of Medicine, New York, NY (G.N., H.O.H.).; Department of Internal Medicine, Turku University Hospital and University of Turku, Finland (T.N.).; Division of Cardiology, Heart and Vascular Institute, Mass General Brigham, Boston, MA (P.T.E., S.J.J., J.R).; Department of Experimental Cardiology, Amsterdam Cardiovascular Sciences, Heart Failure & Arrhythmias, Amsterdam UMC, University of Amsterdam, the Netherlands (D.S.Z., S.J.J.).

**Keywords:** arrhythmias, cardiac, atrial fibrillation, bradycardia, tachycardia

## Abstract

**BACKGROUND:**

Atrial fibrillation and other cardiac arrhythmias pose a major public health burden, but prevention remains difficult. We investigated a genetic variant that we found to act like a natural lifelong cardiac sodium channel blockade.

**METHODS:**

We studied the impact of the Finnish-enriched *SCN5A* missense variant (rs45620037 [T220I]) on cardiac arrhythmias, associated mortality, and ECG phenotypes in a multicohort observational study with >1 million individuals across 3 cohorts (FinnGen, UK Biobank, and Health 2000).

**RESULTS:**

We identified protective effects of T220I on multiple common cardiac arrhythmias, most notably atrial fibrillation (cause-specific hazard ratio [HR], 0.56 [95% CI, 0.50–0.63]; *P*<0.0001), but also ventricular premature depolarization or ventricular tachycardia, as well as increasing susceptibility to conduction-slowing conditions, such as sick sinus syndrome (mostly in older age groups). Overall, T220I conveyed protection from death resulting from cardiac arrhythmia (HR, 0.65 [95% CI, 0.46–0.92]; *P*=0.015) without a significant effect on overall mortality risk (HR, 0.92; *P*=0.27). T220I heterozygosity had similar electrophysiological effects as some pharmacological sodium channel blockers, such as significantly shortening QT intervals (−7.49 ms [95% CI, −10.07 to −4.91] ms; *P*=0.0037; n=3188) in the Health 2000 cohort, which we replicated in the UK Biobank (n=66 616). In addition, T220I protected from (left) heart failure and dilated cardiomyopathy. After myocardial infarction, we found that T220I increased mortality risk, consistent with known sodium channel blocker effects, which, however, normalized to baseline 10 to 15 years after myocardial infarction. We found that T220I could lower a high genetic burden (ie, a high polygenic score) for atrial fibrillation to population average.

**CONCLUSIONS:**

The *SCN5A* T220I variant, consistent with a previously described weak loss-of-function effect, acted like a genetic proxy for cardiac sodium channel blockade. This enabled us to gain new potentially clinically relevant insights for pharmacological sodium channel blockade, such as after myocardial infarction, which would be too risky to investigate with clinical trials. Our findings may also inspire redesign of cardiac sodium channel blockers.

**Clinical Perspective**

**What Is New?**

- The rare genetic variant T220I in the *SCN5A* gene emulates a lifelong cardiac sodium channel blocker as evidenced by its specific electrophysiological and clinical phenotype.
- In the general population, T220I is associated with a reduced risk of tachyarrhythmias, such as atrial fibrillation; a reduced risk of heart failure; an increased risk of bradyarrhythmias; and an overall reduced risk of mortality from cardiac arrhythmias.
- T220I lowers the lifetime risk of atrial fibrillation in individuals with high genetic liability to a risk comparable to that of the general population.
- In individuals with a recent myocardial infarction, T220I is associated with an initial increase in mortality risk in the first 5 years after infarction.

**What Are the Clinical Implications?**

- T220I may be a useful genetic proxy for predicting the benefits and side effects of sodium channel blockade, including situations in which clinical trials might be considered too risky.
- T220I protecting against heart failure is of value in the ongoing debate regarding the safety and benefits of sodium channel blockade for cardiac rhythm control.
- Our data are consistent with a balanced risk–benefit profile of long-term sodium channel blockade in the general population, but also with harm from sodium channel blockade after a recent myocardial infarction.
- T220I modulates the associations of a polygenic score for atrial fibrillation and can counterbalance the effects of a high polygenic risk (genetic liability) for atrial fibrillation.

Cardiac arrhythmias pose a major health burden. Atrial fibrillation (AFib) is the most common arrhythmia, with >4 million incident cases and 300 000 deaths per year globally.^1^ AFib increases the risk for many cardiovascular outcomes, including heart failure and stroke, and is associated with increased all-cause mortality risk. However, prevention and control of AFib and its sequelae remain difficult, partially because of heterogeneous causal mechanisms. Both common and rare genetic factors have been described to have large influences on the development of cardiac arrhythmias. AFib has been shown to be highly polygenic.^2^

Ion channels play a large role in the development of cardiac arrhythmias because they are essential for cardiac signaling. The *SCN5A* gene encodes Na_V_1.5, the pore-forming subunit of the cardiac sodium channel responsible for the initiation and propagation of cardiac action potentials. Thus, genetic variants within *SCN5A* can increase the risk for certain cardiac arrhythmias and arrhythmia syndromes, such as Brugada syndrome (BrS)^3,4^ and AFib.^2,5–7^

The different types of clinical presentations caused by *SCN5A* sequence variations can largely be explained by the degree and specific type of sodium channel dysfunction at the molecular level. Loss of function (LoF) of Na_V_1.5 (such as caused by protein truncating variants) is a main cause of BrS,^3^ as well as conduction deficits resulting in bradycardia, conduction blocks, or sick sinus syndrome (SSS). In contrast, gain of channel function has been linked to other specific disease entities, such as long QT syndrome.^3,8,9^ More severe phenotypes, such as congenital SSS, can be caused by more severe LoF of the channel, for instance through the presence of 2 defective *SCN5A* alleles.^10–12^ Genetically caused *SCN5A* LoF is comparable to sodium channel blocker medications, which are used to mediate a desirable LoF effect, but may also increase risk of cardiac arrhythmias in certain contexts.

In a recent study of 180 000 Finnish individuals,^13^ we discovered that carriers of 1 genetic missense variant in *SCN5A*, T220I (rs45620037 or NM_000335.5[SCN5A]:c.659C>T [p.T220I]), had only half the risk of AFib compared with the general population. The T220I variant is 5-fold enriched in the Finnish population to an allele frequency of 0.46%, compared with a frequency of 0.11% in the UK Biobank (UKBB)^14^ and 0.086% in other non-Finnish European populations.^15^ The clinical significance of T220I remains uncertain because of limited data from family studies and case reports^11,16,17^ (for an overview, see Table S1). T220I was previously described in individuals with childhood-onset SSS who also carried a LoF variant on the other allele^18^; however, the evidence was confined to a few families.^19–21^ Because of the high population frequency and absence of large effects on disease, it was classified as a likely benign variant (5 independent submissions) or variant of uncertain significance (4 independent submissions) in ClinVar, a public variant database.^22,23^ An association of T220I with BrS could not be established.^24,25^ Electrophysiological studies revealed weak LoF,^19,20^ characterized by a stabilized inactivation state that reduced sodium current. This impairs the ability of the sinus node to reach the threshold for action potential initiation, thereby diminishing electrical signaling in the heart and thus potentially resulting in bradyarrhythmic effects.^26,27^ The effects of T220I on slowing conduction are in line with a decreased risk for diseases associated with reentry, such as AFib, which aligns with our earlier observations.^13^

Because of the previously established mild LoF effect, we hypothesized that T220I acts as a proxy for a mild sodium channel blocker. We thus hypothesized that T220I should affect cardiac conduction reflected in specific ECG changes. In addition, T220I should affect risk for specific cardiac diseases, such as protecting from AFib (as observed earlier), but also possibly increasing the risk of sinus bradycardias, given that bradycardia is a common side effect of cardiac sodium channel blockers and because the variant was previously associated with SSS.^21,22^ In addition, we hypothesized a potential increased risk for other possible side effects of cardiac sodium channel blockers in T220I carriers, such as conduction blocks, ventricular fibrillation, BrS-like changes, or Torsades de pointes. We further hypothesized that because of its low global allele frequency, T220I might act independently of a polygenic score (PGS) and thus potentially counterbalance an otherwise high genetic burden for AFib. To investigate the effects of T220I on the risk of different cardiac phenotypes, including mortality and conduction parameters measured by ECG, we leveraged data from 3 mostly population-based research cohorts (FinnGen,^28^ the UKBB,^14^ and the Finnish cohort Health 2000 [H2000])^29^ using data from a total of 1 028 508 individuals. The primary analysis was conducted within FinnGen, including longitudinal electronic health record data covering up to 5 decades in >520 000 individuals,^28^ a cohort nearly 3 times larger than when we first discovered the protective effect of the variant.^13^

## METHODS

### Data Availability

Based on national and European regulations (ie, General Data Protection Regulation), access to individual-level sensitive health data must be approved by national authorities for specific research projects and for specifically listed and approved researchers.

FinnGen data can be accessed through the Finnish Biobank FinBB portal (www.finbb.fi; email info.fingenious@finbb.fi). The procedures for access to the H2000 data are described at https://thl.fi/en/research-and-development/thl-biobank/for-researchers/application-process. The procedures for access to the UKBB data are described at https://www.ukbiobank.ac.uk/enable-your-research. See https://finngen.gitbook.io/documentation for a detailed description of data production and analysis, including the code that was used to run analyses, and https://github.com/FINNGEN for further code repositories that were used to run analyses in FinnGen. The polygenic risk score–continuous shrinkage (PRS-CS) pipeline used in FinnGen is detailed at https://github.com/finngen/cs-prs-pipeline. The R code to reproduce figures is available at https://github.com/waseju/scn5a.

### Study Design and Cohorts

We conducted this study using 3 primary cohorts: FinnGen (n=520 210), the UKBB (n=502 250), and H2000 (n=6048). Both the UKBB and H2000 are population-based research cohorts including genetic and health data, initiated in 2006 and 2000, respectively. The FinnGen cohort is a longitudinal research cohort including genetic and health data from national health registers. Follow-up for FinnGen started in 1953, with an end of follow-up in 2023, with a mean follow-up time of 60.81 years. FinnGen was used as the primary discovery cohort for investigating the effects of T220I on cardiac disease. Further details about FinnGen, including genotyping of T220I and other variants, can be found in the FinnGen flagship article.^28^ The UKBB is a prospective study cohort of 500 000 participants from the United Kingdom between 40 and 69 years of age at recruitment; individuals were recruited from 2006 through 2010, with a mean age at the end of follow-up of 64.3 years.

Details on genotyping and Hardy-Weinberg equilibrium of T220I are available in previous articles^14,28^ and Note S1. We restricted the analyses to individuals with European ancestry. Further details about exclusion criteria are shown in Note S1 and Figures S1 through S3.

**Figure 1.**
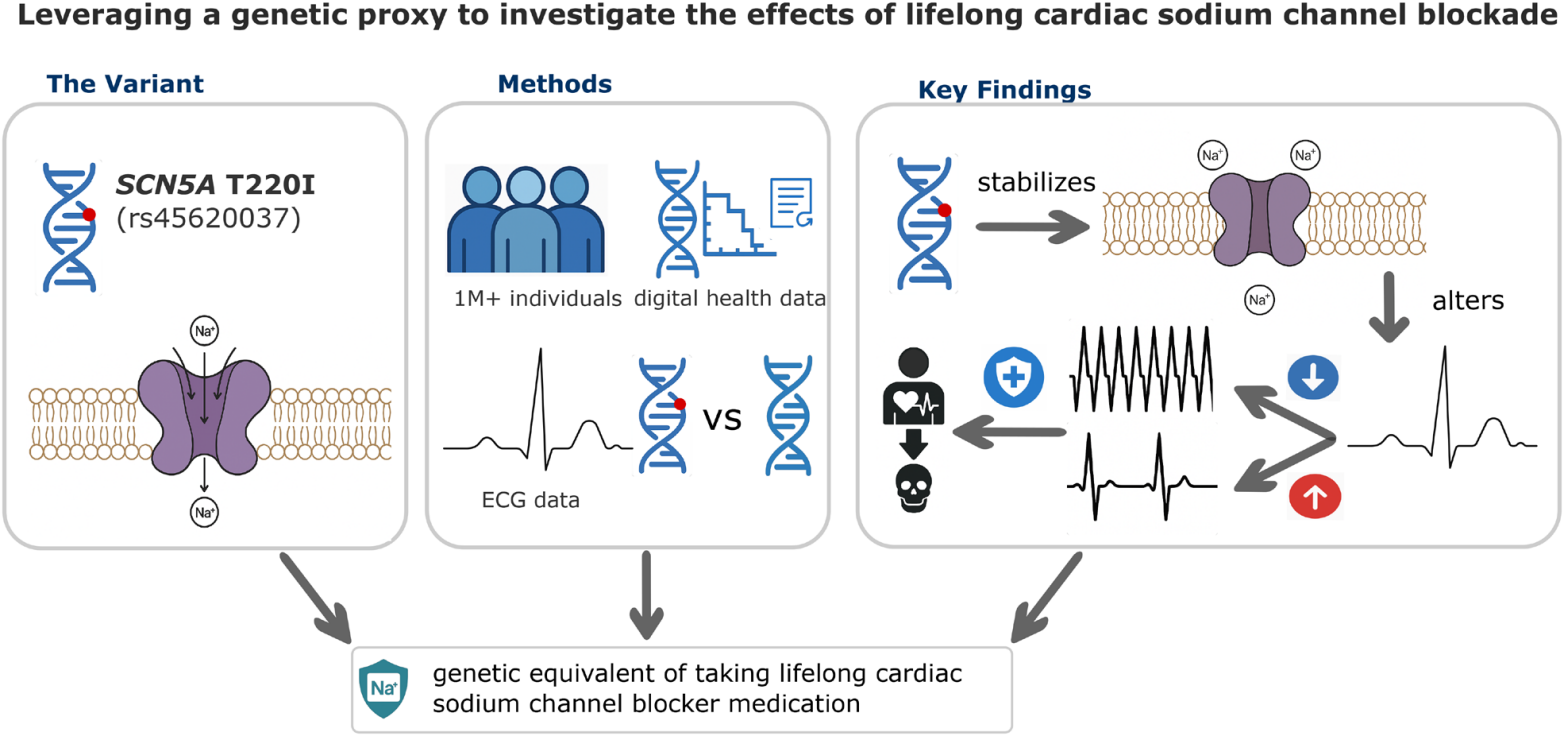
**T220I acts as a genetic equivalent of a lifelong cardiac sodium channel blocker.** We used genetic, electronic health record, and ECG data to show the substantial protection of T220I against tachyarrhythmias, with increased risk for bradyarrhythmias, and overall protection against cardiac arrhythmia–related death.

An overview of the study design is shown in Figure 1. Analysis workflow is shown in Figure S4. Descriptive statistics on all cohorts are presented in Table 1. We obtained ethics approvals from the respective institutional review boards, and all participants provided informed consent for biobank-based research. Our study design is in agreement with STROBE (Strengthening the Reporting of Observational Studies in Epidemiology) requirements.^30^

**Table 1.**
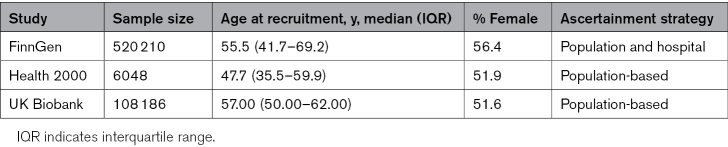
Details of the Participating Biobanks

### Phenotype Definitions

We studied the effect of T220I on cardiac disease phenotypes in FinnGen and validated associations in the UKBB. For a list of phenotypes, see Table S2. We studied the effect of T220I on ECG phenotypes in H2000 and the UKBB. We defined cardiac arrhythmias, myopathies, and heart failure, including AFib and BrS, using ICD (International Classification of Diseases) codes I40 through I50 and their subcategories (I40.[0–9] to I50.[0–9]) from national health care registries in FinnGen, with 1.87 million cardiac arrhythmias (I44.[0–9] to I49.[0–9]) recorded across the cohort, affecting 95 519 unique individuals, and 9771 deaths resulting from cardiac arrhythmias, as documented in national death registries. Case numbers for cardiac arrhythmias with significant associations with T220I carrier status are shown in Table 2.

**Table 2.**
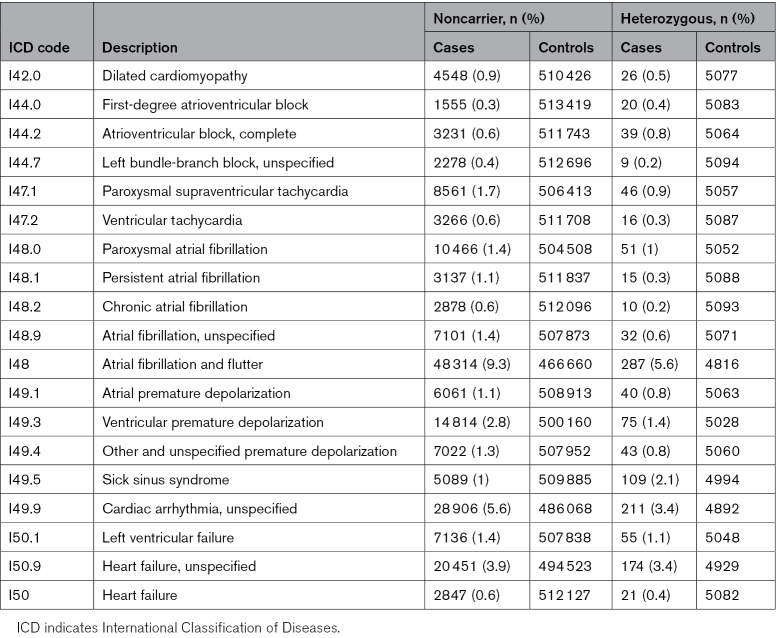
Case and Control Counts of Noncarriers and T220I Heterozygotes for Nominally Significant Effects of T220I Heterozygosity on Disease in FinnGen

For a flowchart of phenotype data quality control, see Figures S1 through S3. To control for potential confounders, we included covariates such as age, sex, body mass index, medication use (including psychotropic drugs, antidepressants, and glaucoma medication), diseases, and other factors affecting heart function; see Table S3 and Note S2 for details about covariates and sensitivity analyses, respectively. For details on automated ECG measure analyses in H2000 and the UKBB, see Note S3. We also visually inspected 62 ECGs of T220I heterozygotes in H2000 and 65 ECGs in the UKBB.

### Statistical Analysis

We performed an association study between the T220I variant and case–control status for ICD-10 categories I40 through I50 and their subcategories (I40.[0–9] to I50.[0–9]) in FinnGen and the UKBB using the PheWAS R package (version 0.99.6-1), with birth year, sex, genotyping array, and first 10 principal components (PCs) of genetic ancestry as covariates. To detect potential nonadditive genetic effects, we analyzed heterozygous and homozygous carriers separately. To correct *P* values for multiple testing of 148 tests, we applied a Bonferroni threshold of 3.37×10^−^^4^.

For subsequent time-to-event analyses, we fitted Cox proportional hazards models (R package survival, version 3.7.0). Follow-up time began at birth and ended at the age at first diagnosis for cases or at the age at the last recorded entry or death, whichever occurred first. We adjusted the models for the first 10 PCs of genetic ancestry, sex, and genotyping array to account for potential technical artifacts. To provide an overview of the effects of the variant across all clinical end points within FinnGen, we conducted an additional exploratory phenome-wide association study investigating the effects of T220I on 2469 phenotypes. The results of this exploratory analysis are visualized in Figure S5.

All statistical tests were 2-sided, without assuming a specific direction of effect. We leveraged comprehensive registry data in which follow-up for each individual began at birth. For death analysis, we used adjusted Cox proportional hazards models integrating age at recruitment. To estimate etiological effects of T220I carrier status, we used cause-specific Cox models for both cardiac phenotypes and arrhythmia-related mortality with all-cause mortality outcome as the competing risk. To quantify absolute risk in the presence of competing risks, we fitted Fine-Gray subdistribution models (R package: tidycmprsk, version 1.24.1) for cardiac phenotypes and death resulting from cardiac arrhythmias, adjusted for genotyping array, first 10 genomic PCs, birth year, and sex. Left-truncated time-to-event analyses used age as the underlying time scale with delayed entry at the age of DNA donation. Post–myocardial infarction (MI) hazard ratios (HRs) were modeled with Cox proportional hazards models and delayed entry at the start of an interval. All survival analyses for incident disease were right-censored at age 80 years. All survival analyses for disease mortality were right-censored at age 100 years.

We tested the effects of T220I on different ECG measures in the UKBB and H2000 using linear regression as implemented in the glm function in R (version 4.3.0). In both cohorts, we corrected ECG measures for heart rate according to Bazett (QTc=QT [ms]/√RR [s]) to adjust for heart rate variability.^31^ In the UKBB, we evaluated the association of T220I with each of 4 electrocardiographic measurements (QRS, PR, QT, and P-wave duration), with genotype-inferred sex, age during the ECG visit, the first 10 genomic PCs, and the genotyping array as covariates.

Within H2000, we included genotype, sex, age, and interaction terms (age×sex, age×age) as independent variables. In H2000, we further adjusted for covariates such as calcium, thyroid-stimulating hormone, systolic and diastolic blood pressure, body mass index, fitness index, illnesses (respiratory, arrhythmia, and heart disease), medication use, and the first 4 PCs of genetic ancestry (see also Note S2).

To capture the genetic liability to AFib, we calculated PGSs for AFib and BrS using PRS-CS,^32^ a Bayesian method that incorporates linkage disequilibrium. To construct disease-specific PGSs, we used publicly available genome-wide association study summary statistics for AFib^33^ and BrS^4^ and standardized scores to a mean of 0 and an SD of 1. To avoid potential linkage disequilibrium effects, not captured by PRS-CS, we excluded genetic variants located ±500 kb around T220I.

To assess whether the protective effect of T220I was additive with an AFib-PGS, we performed an interaction analysis with the survival package between T220I carrier status and the PGS, treating the PGS as a continuous variable.

We further categorized individuals into disease-specific risk bins: high PGS (>1 SD above the mean), low PGS (< −1 SD below the mean), and average PGS (between −1 SD and 1 SD), and conducted survival analysis across the lifetime using our usual covariates in addition to PGS bins. All analyses were performed in R 4.3.0.

### Biases

To adjust for population stratification, batch effects, and changes in disease definitions across time, we corrected all models for PCs of genetic ancestry, sex, genotyping array, and year of birth. We restricted analyses to either European British or Finnish ancestry within each cohort, respectively, to reduce confounding attributable to ancestry. Sensitivity analyses demonstrated that the findings remained stable after accounting for medication use, competing risks due to other causes of death, and potential left‐truncation bias (Notes S2 and S4).

### Ethics and Data Approval

This study complies with all relevant ethical regulations. We obtained approval for the FinnGen study protocol from the Ethics Committee of the Hospital District of Helsinki and Uusimaa (approval HUS/990/2017). For the UKBB, ethical approval had been granted by the National Information Governance Board for Health and Social Care and the NHS North West Multicenter Research Ethics Committee.

All participants provided informed consent through electronic signature at the baseline assessment. The data used in this study are available in the UKBB database under application numbers 77717 and 17488, 17488 was approved by the local Massachusetts General Hospital Institutional Review Board.

Data are available in a public, open access repository (https://www.ukbiobank.ac.uk). For further details about the ethics and data approval, see Note S5.

## RESULTS

### Description of Cohorts

We set out to test the effects of T220I on lifetime risk of cardiac diseases using 3 different cohorts: FinnGen, the UKBB, and H2000. We used FinnGen (data freeze 12) as our main discovery cohort, which included 520 210 individuals. The allele frequency of T220I was 0.5% in FinnGen, with 28 homozygotes and 5021 heterozygotes; 0.51% in the H2000 study, with 62 heterozygotes and no homozygotes; and 0.10% in the UKBB, with 820 heterozygotes and no homozygotes. Further descriptive statistics of the 3 cohorts are shown in Table 1.

### The Effect of T220I on Common Cardiac Arrhythmias

Because T220I was previously found to cause mild LoF of the Na_V_1.5 channel, it may be a genetic proxy for a lifelong mild sodium channel blockade. We thus explored the effect of T220I on diseases associated with sodium channel blockade. We tested whether heterozygotes of T220I are associated with cardiac arrhythmias and heart failure, testing cardiac-related ICD codes I40 through I50 within FinnGen.

**Figure 2.**
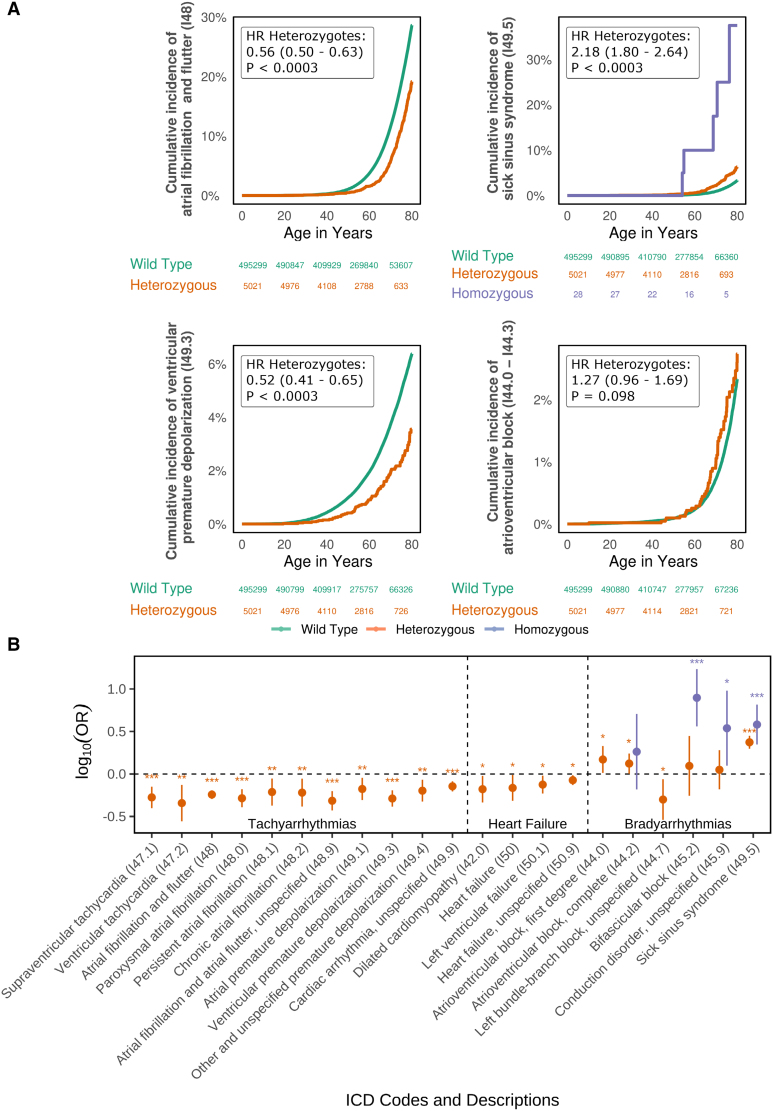
**Effect of the *SCN5A* variant T220I on specific cardiac arrhythmias across the lifetime and for incident cases. A**, Age at first diagnosis (*x* axis) of different cardiac arrhythmias, separately for different T220I carrier statuses. Cumulative disease incidence is given on the y *axis*. Hazard ratios (HRs) of the effect of T220I across the lifetime with 95% CIs and respective *P* values are shown. T220I homozygotes are in blue; T220I heterozygotes, in orange; and wild-types, in green. **B**, log_10_ of odds ratios (ORs) of significant associations of T220I with cardiac phenotypes I40 through I50. Statistical significance before multiple testing correction is denoted by asterisks: *P<0.05, **P<0.01, ***P<0.001. Homozygous carrier states are in blue; heterozygotes, in orange. ICD indicates International Classification of Diseases.

We found that in a heterozygous state, T220I was associated with protection from multiple types of tachycardia, such as AFib (odds ratio [OR], 0.57 [95% CI, 0.51–0.64]; *P*<0.0003), which we replicated in the UKBB (Note S6), or ventricular premature depolarization (Figure 2B). However, it also increased risk of cardiac bradyarrhythmias, such as SSS (OR, 2.36 [95% CI, 1.98–2.80]; *P*<0.0003; Figure 2B). Disease associations were relatively unaffected by the removal of related individuals. Cases and controls are shown in Table 2. We also found a protective effect of T220I heterozygosity on ventricular tachycardia (OR, 0.45 [95% CI, 0.28–0.74]; *P*=0.002) that we did not necessarily expect considering the known side effects of long-term sodium channel blockade for the prevention of AFib. Further sensitivity analyses with Cox proportional hazards models revealed that the protective effects were not attributable to an earlier age at disease onset or confounded by use of medications (Note S4). In homozygotes, we found an increased risk for bradyarrhythmias, specifically bifascicular block (OR, 7.88 [95% CI, 3.63–17.11]; *P*<0.0003) and SSS (OR, 3.81 [95% CI, 2.21–6.55]; *P*<0.0003; Table S4).

Next, we explored the effects of T220I on cardiac diseases across the lifetime. Most effects remained consistent across the life course (Figure 2A). Heterozygotes were protected against cardiac arrhythmias throughout their lifetime, but were also at an increased risk of bradyarrhythmias, such as SSS and atrioventricular block; survival curves are shown in Figure 2A.

Consistent with this, we found a higher rate of pacemaker procedures in T220I heterozygotes (HR, 1.35 [95% CI, 1.14–1.59]; *P*<0.0003) and homozygotes (HR, 5.9 [95% CI, 2.21–15.8]; *P*=0.0047; Figure S6).

Exploring the effect of T220I on heart failure and cardiomyopathies, we found that T220I nominally protected against dilated cardiomyopathy (HR, 0.66 [95% CI, 0.46–0.94]; *P*=0.021) and heart failure (HR, 0.84 [95% CI, 0.75–0.93]; *P*=0.0012) across the lifetime.

### T220I Protects From Death Resulting From Cardiac Arrhythmias

**Figure 3.**
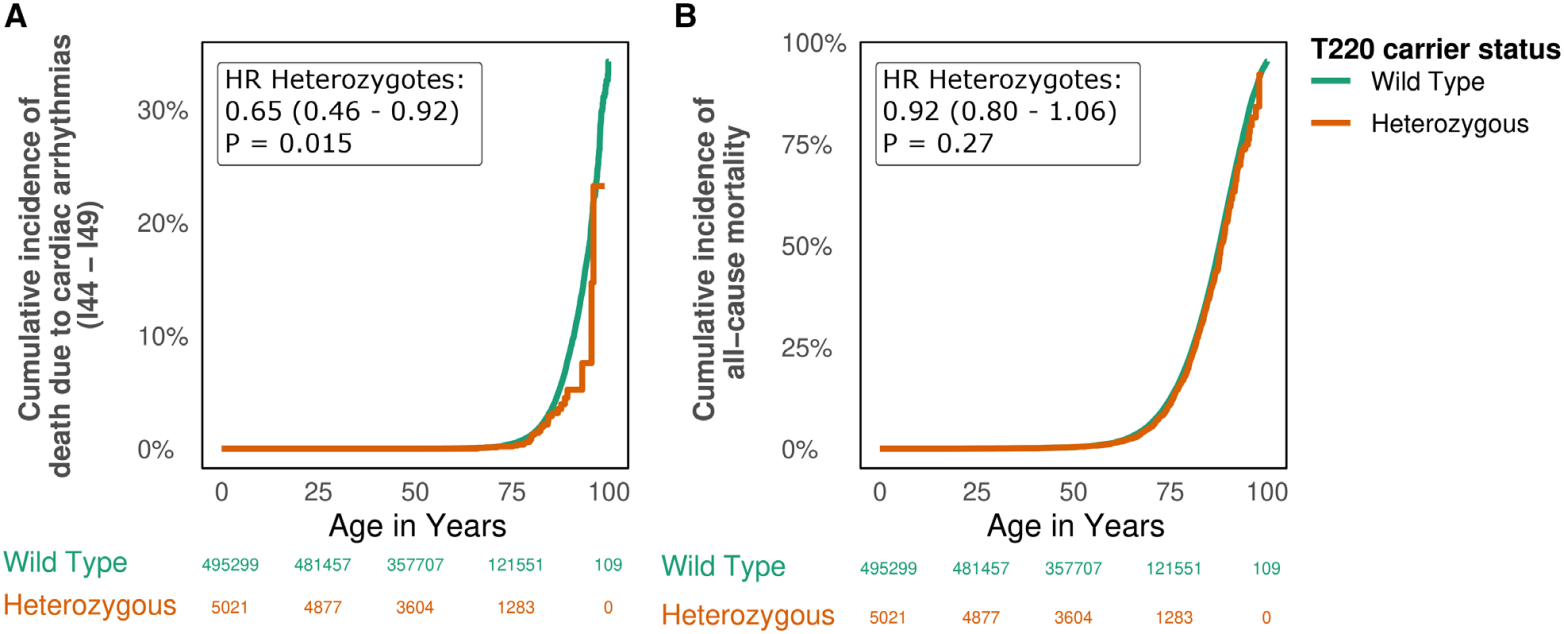
**T220I protects from death** resulting from **cardiac arrhythmias with no effect on all-cause mortality. A**, Cardiac arrhythmias. **B**, All-cause mortality. Data are shown as cumulative incidences (*y* axis) across time (age in years, *x* axis). Significant hazard ratios (HRs) across the lifetime are shown inside the survival plots. Heterozygotes are in orange; wild-types, in green.

Because T220I decreased but also increased risk for multiple cardiac arrhythmias, we wanted to explore whether T220I had a net positive or negative overall effect on death resulting from cardiac arrhythmias across the lifetime. Overall, we found a protective effect against death resulting from cardiac arrhythmia (HR, 0.65 [95% CI, 0.46–0.92]; *P*=0.015; Figure 3). This effect was likely mainly driven by the protective effect of T220I against death resulting from AFib (HR, 0.58 [95% CI, 0.37–0.85]; *P*=0.0068). We found no effect on overall cardiovascular mortality (HR, 0.99 [95% CI, 0.85–1.15]; *P*=0.90) or all-cause mortality (HR, 0.92 [95% CI, 0.80–1.06]; *P*=0.27). This was expected because we found no effect of T220I on any diseases not originating in cardiac cells (for an exploratory analysis, see Figure S5). After adjusting for competing risks attributable to other causes of death, we still observed the protective effect of T220I on death resulting from cardiac arrhythmias (subdistribution HR, 0.67 [95% CI, 0.46–0.97]; *P*=0.032, method: Fine-Gray model) and other cardiac phenotypes, such as AFib (subdistribution HR, 0.57 [95% CI, 0.51–0.63]; *P*<0.0003); for left-truncation and competing risk-adjusted cumulative incidence curves, see Figure S7.

### Associations of T220I With Outcomes After MI

We next sought to investigate the effect of T220I in individuals with structural heart disease, motivated by the finding of increased mortality in patients who received sodium channel blockers after MI in CAST (Cardiac Arrhythmia Suppression Trial).^34^ In 31 492 individuals who had an MI, we found that 307 T220I carriers were still protected from AFib (HR, 0.64 [95% CI, 0.44–0.93]; *P*=0.019). After MI, the protective effect of T220I from cardiac arrhythmia–related mortality stayed similar, although it was not significant because of the reduced sample size (HR, 0.43 [95% CI, 0.15–1.14]; *P*=0.091), with no effects on all-cause mortality (HR, 1.14 [95% CI, 0.94–1.38]; *P*=0.17) or cardiovascular mortality (HR, 1.01 [95% CI, 0.83–1.22]; *P*=0.94). However, when restricting our observation period to 2 years after MI to simulate CAST, we also found increased all-cause mortality in the group of T220I carriers, similar to pharmacological sodium channel blockade, up to 5 years after MI (HR, 2.34 [95% CI, 1.23–4.43]; *P*=0.009). When we examined individuals who survived longer after MI, the T220I-associated mortality risk declined continuously, reaching baseline 15 years after MI (Figure S8; Note S7).

### T220I Alters the Effects of Common Genetic Variation on AFib

**Figure 4.**
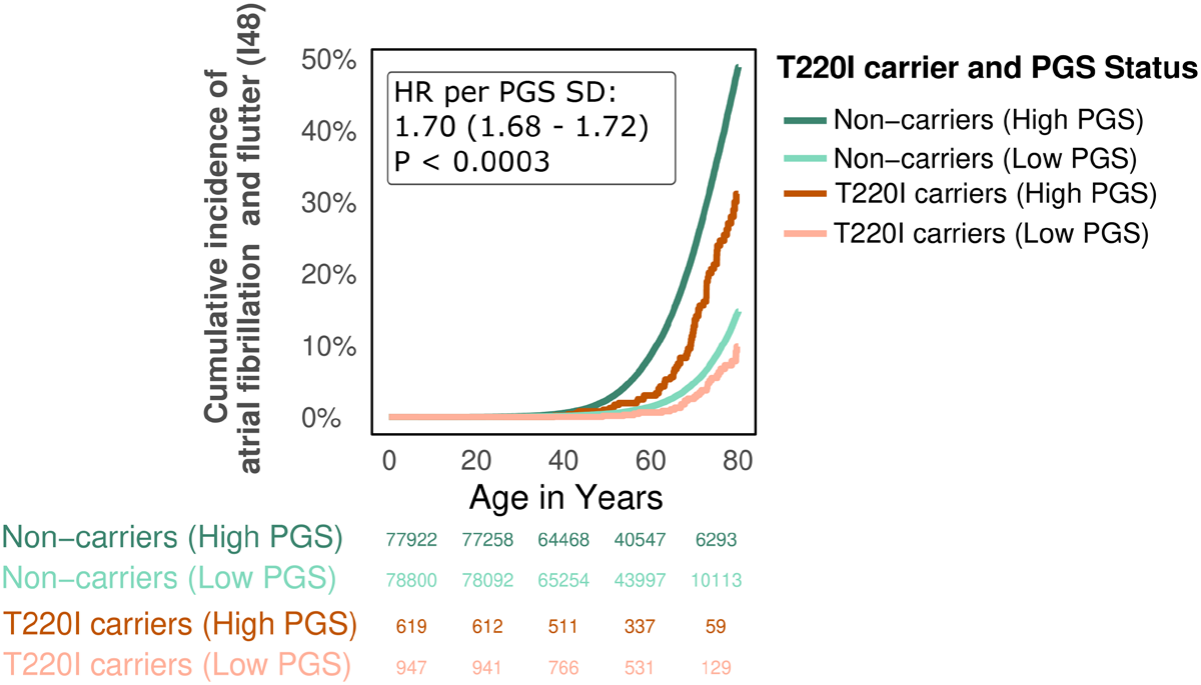
**T220I reduces a high genetic risk for atrial fibrillation.** Age at first diagnosis of atrial fibrillation (AFib), stratified by carrier status of T220I and high or low AFib–polygenic score (PGS). Data are shown as cumulative incidence of AFib (*y* axis) across the lifetime (age in years, *x* axis). Hazard ratio (HR) of AFib-PGS effect across the lifetime is shown in the figure. T220I heterozygotes are in orange; wild-types, in green. Low PGS indicates individuals with AFib-PGS < −1 SD. High PGS indicates individuals with AFib-PGS > +1 SD.

Next, we wanted to test how T220I influences the effect of common genetic variation on AFib. Thus, we computed a disease-specific PGS for AFib (method: PRS-CS^32^) and tested its association with AFib risk in relation to T220I carrier status in FinnGen. As expected, higher AFib-PGS increased risk for AFib across the lifetime (HR per SD AFib-PGS increase, 1.70 [95% CI, 1.68–1.72]; *P*<0.0003), in line with previous studies.^35^ However, T220I could counterbalance the effect of a high AFib-PGS on AFib risk. For example, individuals with a high AFib-PGS (PGS > +1 SD) who also carried T220I had an average risk for AFib across the lifetime (Figure 4). This association remained significant after the removal of all variants within ±500 Kb of T220I from the PGS to exclude potential variants that could capture the effect of T220I resulting from linkage disequilibrium. We found that the variant significantly modulated how common genetic variation affects AFib risk (attenuation factor, 0.88 [95% CI, 0.81–0.99]; *P*_interaction_=0.017; PGS HR per SD in variant carriers, 1.50 [95% CI, 1.36–1.67]; *P*<0.0003), which we could not attribute to sex or age interactions (Figure S9A and S9B).

### T220I Is Associated With Shortened QT and PR Intervals

**Figure 5.**
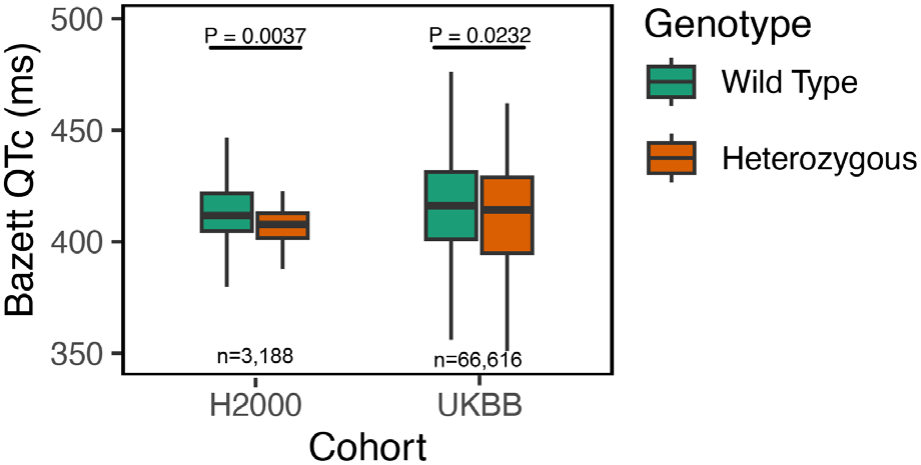
**Bazett-corrected QT intervals for heterozygotes and wild-types of T220I.** Data from the Health 2000 (H2000) study (n=3188) and the full UK Biobank (UKBB) data set (12-lead resting or 3-lead upright ECG before exercise test; n=66 616) are shown as boxplots. Boxplots show the median and interquartile range with whiskers extending to 1.5×interquartile range. Outliers omitted for clarity. Significantly shortened corrected QT intervals (QTc) were found in both cohorts (*P*=0.0037 for H2000 and *P*=0.023 for the UKBB).

We evaluated how the T220I variant affects cardiac conduction, investigating 5 ECG measures. We found significantly shortened QT intervals (QTc, Bazett-corrected) in the H2000 cohort (−7.49 ms [95% CI, −10.07 ms to −4.91 ms]; *P*=0.0037; method: linear regression; n=3188) and in the UKBB after the exclusion of participants with prolonged QRS duration or extreme heart rates (−4.41 ms [95% CI, −8.22 to −0.60 ms]; *P*=0.023; n=66 616; Figure 5). Effect sizes of T220I on QTc time remained similar in a smaller data set including only 12-lead ECGs in the UKBB (−4.95 ms [95% CI, −10.29 to 0.39 ms]; *P*=0.069). The associations in H2000 also remained significant when using a different QT correction method (Framingham^36^) or adjusting for effects of medications, fitness measures, and blood pressure (see sensitivity analyses in Note S2). We also found associations with PR time in both the 12-lead (11.98 ms [95% CI, 5.86–18.10]; *P*<0.001) and the combined 12-lead and 3-lead ECG data sets (6.15 ms [95% CI, 1.98–10.31]; *P*=0.0038) in the UKBB (Table S5). In H2000, we found no effect on PR time. In addition, we searched for abnormalities in real ECGs of T220I carriers in the H2000 cohort (n=62) and the UKBB (n=65). We found no Brugada-like patterns in the H2000 cohort or the UKBB, but a high number of sinus bradycardia cases in the H2000 cohort, in line with increased bradycardia-associated diseases in variant carriers in FinnGen.

## DISCUSSION

The Finnish-enriched *SCN5A* variant T220I is notable for its relatively large protective effect against AFib, with carriers having about half the relative disease risk of wild-type individuals, which is much larger than commonly observed in genome-wide association studies. To our knowledge, no similarly sized protective effects against cardiac arrhythmias or heart failure have been described for any other variants in *SCN5A* or in other genes. Here, we characterized the effect of T220I on cardiac phenotypes and conduction measures in >1 million individuals. In agreement with the experimental evidence of a mild LoF effect, our results are consistent with the variant acting similarly to a sodium channel blocker. While protecting against multiple cardiac arrhythmias, such as AFib and heart failure, T220I also conferred increased risks consistent with typical sodium channel blocker–associated side effects, including increased risk for bradycardia and slowed conduction and specific ECG changes. Overall, the protective effects predominated, resulting in an overall protection against death resulting from cardiac arrhythmias. We further showed that the variant can effectively reduce the genetic liability for AFib conferred by common variants from a high to an average risk.

### Our Data Mostly Confirm Previous Associations of T220I With Cardiac Arrhythmias

The T220I variant has been previously implicated in different cardiac diseases, based on studies of individual families^11,18,20,24^ (for an overview, see also Table S1). Here, we critically evaluated these previous findings in several large cohorts of a total of >1 million individuals across 3 biobanks. We confirmed our earlier observation of the protective effect of T220I against AFib^13^ with an almost tripled cohort size.

T220I has been observed in individuals with childhood-onset SSS in one family, but only together with a larger LoF effect variant on the other allele, in a compound heterozygous state.^19^ Here, we observed an elevated risk for SSS in T220I, particularly for homozygotes, mostly in adult age groups, classifying the variant as a risk factor^37^ for adult-onset SSS. Therefore, it could also act as a hypomorphic variant contributing to a more severe childhood-onset SSS when in a compound heterozygous state with a LoF variant, in agreement with its experimentally found mild LoF effect,^11,19^ although we did not provide direct evidence in this study. With our data, we cannot confirm previous case reports stating that T220I may increase risk for AFib or dilated cardiomyopathy, and rather provide evidence of protective effects on these outcomes. Given that the earlier studies were confined to individual families, it is much more likely that these were chance observations, whereas our study was powered to investigate such disease associations.

### Protection and Risk Increases for Specific Arrhythmias Implies Both Similarities and Differences Between T220I and Known Effects of Specific Sodium Channel Blockers

Given the mild LoF effect of T220I,^17,18^ it may act similarly to a lifelong sodium channel blocker. Sodium channel blockers are a class of drugs (Vaughan Williams class 1 antiarrhythmics) that are often prescribed to control atrial and ventricular cardiac arrhythmias. Their common side effect is sinus bradycardia,^38^ slowing of the heart rate through the sinoatrial node, as well as cardiac conduction delays or blocks. This aligns with our observations of an increased rate of SSS, atrioventricular block, and bifascicular blocks in variant carriers in homozygotes and partially or to a lesser degree in heterozygotes. These findings are also consistent with hypomorphic *SCN5A* variants being associated with conduction disorders.^5,6,18^ Other known side effects of class 1 antiarrhythmic sodium channel blockers are atrial flutter, ventricular tachycardia, ventricular fibrillation, Torsades de pointes, and Brugada-like patterns. Because some of these conditions, such as ventricular fibrillation, can be directly life-threatening, these side effects have limited the use of sodium channel blockers for AFib. We were surprised to find no increased risk for these conditions in carriers of T220I. On the contrary, we found protective effects of T220I from ventricular tachycardia. Different mechanisms could explain the absence of common side effects of sodium channel blockers. One reason may lie in highly specific effect of T220I on cardiac sodium channels, whereas common sodium channel blocker drugs, such as quinidine, have more nonspecific effects, such as additional blockage of potassium channels.^39^ However, increased specificity cannot be the only explanation, considering that BrS caused by *SCN5A* LoF and consequently lower Na_V_1.5 expression levels is associated with a highly elevated risk (up to 30%) of ventricular fibrillation or ventricular tachycardia.^40^ A possible explanation could be a dosage effect, with Na_V_1.5 blockade conferring protection from AFib and not causing ventricular tachycardia within a certain therapeutic window. T220I may have such protective effects also in a homozygous state within that therapeutic window, as we did not observe any case of ventricular fibrillation among 28 T220I homozygotes. In addition, we and others^24^ did not observe any Brugada-like ECG patterns in T220I carriers. Another explanation could lie in the specific mechanism of the variant. From electrophysiology experiments, T220I is thought to stabilize the inactivated state of the cardiac sodium channel through a gating pore current.^27^ Because of its location in the voltage sensor of the channel, which is key for channel response to voltage changes,^27^ T220I was previously thought to open an abnormal Na+ permeation pathway (also called “gating pore”). The altered channel morphology should cause a leakage of Na+ ions during the resting phase of the channel, allowing Na+ to flow into the cardiac cells during diastole of the heart.^27^ This may cause a partial depolarization of the hyperpolarized resting membrane potential stabilizing the inactivated state of the sodium channels. As a result, fewer sodium channels are thought to be available during action potential firing,^26,27^ thus reducing the peak sodium current and slowing the conduction velocity.^19,20^ This could have beneficial effects in reducing late sodium current, in line with our observations of shortened QT intervals in T220I carriers. Regarding the effects on QT time, T220I resembles the effects of the weaker class 1B sodium channel blockers, such as lidocaine, which are used to treat ventricular tachycardia in emergency situations. Similarly to lidocaine, T220I also lacks an effect on QRS and P-wave duration. However, a possible prolonged PR interval in T220I carriers is not a known typical effect of lidocaine.^39^ In addition, lidocaine is not known to confer protection from chronic AFib, heart failure, or dilated cardiomyopathy. The molecular mechanisms thus seem somewhat different between class 1B sodium channel blockers and T220I. Precise mechanisms of T220I can only be clarified by more detailed experimental investigations.

In addition to protection from cardiac arrhythmias, we found protective effects of T220I from dilated cardiomyopathy and (left) heart failure (with nominal significance individually, but consistently in the same direction). This is particularly interesting because medical rhythm control in AFib is generally thought to not improve outcomes over rate control, potentially because of toxic side effects of antiarrhythmic drugs.^41,42^ However, more recent evidence suggests a potential positive effect of rhythm control in early AFib,^43^ a potential paradigm shift in the field^43^ in line with our observations. Whereas AFib prevention may play a role in protecting T220I variant carriers from heart failure, other mechanisms, such as T220I protection against premature ventricular depolarization, might also contribute. T220I gives us a unique opportunity to investigate the potential risks and benefits of sodium channel blockade in specific scenarios.

After the surprising finding of elevated mortality resulting from cardiac arrhythmias in patients who received sodium channel blockers after an MI in CAST,^34^ sodium channel blockers have generally been considered to be contraindicated in individuals with structural heart disease (MI or heart failure) over the past 30 years. We also observed elevated all-cause mortality in patients with “genetic sodium channel blockade” (T220I carriers) after MI, but T220I-associated mortality continuously decreased 10 to 15 years after MI. This is a promising starting point to motivate evaluation of non-class 1c sodium channel blockers in specific patient populations, in whom new clinical trials would otherwise be considered too risky or unethical.

In our study, T220I could counterbalance a high disease-specific PGS for AFib, capturing genetic liability for AFib conferred by common variants. Individuals with high AFib-PGS who carried T220I had only an average lifetime risk of AFib. As such, T220I carrier status may represent an important modifier in personalized prediction models for AFib.

### Limitations

Our study has several limitations. First, although 28 homozygotes represents a sizable number for a rare variant study, this sample size limits the power to detect effects on rarer phenotypes. However, given the dosage effects we observed for well-powered phenotypes, extrapolating effects from heterozygotes is likely to provide good approximation. Second, our clinical information in FinnGen is restricted to ICD codes, as we did not have access to detailed clinical notes. Third, because our analyses were restricted to participants of European ancestry, further studies will be useful to assess the generalizability of our findings.

### Conclusions

In this study, we found that the genetic *SCN5A* T220I variant acts like a lifelong sodium channel blocker, mostly providing protection from diverse cardiac arrhythmias, while also increasing risk of bradyarrhythmias, similar to sodium channel blocker side effects. Overall, T220I had a net protective effect on mortality resulting from cardiac arrhythmia. We could use this genetic proxy to learn about potential applications of pharmacological sodium channel blockade. For example, the finding that T220I is protective against heart failure contributes to an ongoing debate in the field. T220I may be useful as a genetic proxy to assess sodium channel blockade in specific populations, such as after MI (similar to CAST), adding further information that would be unethical to investigate with clinical studies. Experimental workup will be imperative to fully understand the precise effects of T220I and the related therapeutic potential.

## ARTICLE INFORMATION

### Acknowledgments

The authors thank Carl Jannes Neuse for carefully reading and commenting on the manuscript; Andrea Eoli for providing feedback on figure visualization; and participants and investigators of the FinnGen study, the UKBB, and H2000 for their contributions. The following biobanks delivered biobank samples to FinnGen: Auria Biobank (www.auria.fi/biopankki), THL Biobank (www.thl.fi/biobank), Helsinki Biobank (www.helsinginbiopankki.fi), Biobank Borealis of Northern Finland (https://www.ppshp.fi/Tutkimus-ja-opetus/Biopankki/Pages/Biobank-Borealis-briefly-in-English.aspx), Finnish Clinical Biobank Tampere (https://www.pirha.fi/web/english/for-professionals/finnish-clinical-biobank-tampere), Biobank of Eastern Finland (www.ita-suomenbiopankki.fi/en), Central Finland Biobank (www.ksshp.fi/fi-FI/Potilaalle/Biopankki), Finnish Red Cross Blood Service Biobank (www.veripalvelu.fi/verenluovutus/biopankkitoiminta), Terveystalo Biobank (www.terveystalo.com/fi/Yritystietoa/Terveystalo-Biopankki/Biopankki), and Arctic Biobank (https://www.oulu.fi/en/university/faculties-and-units/faculty-medicine/northern-finland-birth-cohorts-and-arctic-biobank). All Finnish Biobanks are members of BBMRI.fi infrastructure (https://www.bbmri-eric.eu/national-nodes/finland).

### Sources of Funding

The FinnGen project is funded by 2 grants from Business Finland (HUS 4685/31/2016 and UH 4386/31/2016) and the following industry partners: AbbVie Inc, AstraZeneca UK Ltd, Biogen MA Inc, Bristol Myers Squibb (and Celgene Corporation & Celgene International II Sàrl), Genentech Inc, Merck Sharp & Dohme LCC, Pfizer Inc, GlaxoSmithKline Intellectual Property Development Ltd, Sanofi US Services Inc, Maze Therapeutics Inc, Janssen Biotech Inc, Novartis AG, and Boehringer Ingelheim International GmbH. This work was supported by the German Research Foundation (DFG; grant 516649954 to Dr Heyne) and the Hasso Plattner Foundation. The funders had no role in study design; data collection, analysis, or interpretation; manuscript preparation; or the decision to submit the manuscript for publication. Dr Ellinor is supported by grants from the National Institutes of Health (RO1HL092577, RO1HL157635, R01HL177209), from the American Heart Association (961045), from the European Union (MAESTRIA 965286), and from the Fondation Leducq (24CVD01).

### Disclosures

Dr Ellinor has received sponsored research support from Bayer AG, Novo Nordisk, Bristol Myers Squibb, and Pfizer, and has consulted for Bayer AG.

### Supplemental Material

Checklist

Notes S1–S7

Figures S1–S9

Tables S1–S5

## Supplementary Material


