# Supplementary material for: Leveraging a Genetic Proxy to Investigate the Effects of Lifelong Cardiac Sodium Channel Blockade
Source: Circulation. 2025 Nov 24;152(24):1679–90. doi: 10.1161/CIRCULATIONAHA.125.075057 (PMC12700700; doi:10.1161/CIRCULATIONAHA.125.075057)
Supplement: Supplementary file 1 [file cir-152-1679-s001.pdf]

SUPPLEMENTAL MATERIAL.

Supplementary Notes

Supplementary Note 1 - Details on phenotype and genotype quality control

Please see Supplementary Figures S1-S3 for flowcharts of data quality control steps (including sample size changes) for different analyses in different cohorts.

We applied the following general exclusion criteria within H2000: older than 90 years, being in poor health with a fitness index < 54, and classified as morbidly obese with a BMI > 40. We also excluded individuals with abnormal blood test results (calcium concentration <2.2 or >2.6 mmol/l, or TSH >4.5 mU/l), extreme blood pressure (diastolic >120 mm Hg or systolic >180 mm Hg), and individuals taking heart medications, including cardiac glycosides, antiarrhythmics, beta blockers, calcium antagonists, and antithrombotic drugs.

For FinnGen, samples were removed if they exhibited a mismatch between genetic and registered sex, high variant missingness (>2%), excessive heterozygosity, or a high degree of relatedness to other samples (π^>0.1). Prior to genomic analyses, we excluded participants whose submitted gender did not match their sex inferred from genotypes, participants who were outliers in heterozygosity, participants with putative sex chromosome aneuploidy, and participants with >5% genotype missingness rate for the UKBB We restricted the analyses to the white British ancestry subset within the UKBB and removed related participants with kinship values > 0.0884. Differences between exclusion criteria were based on available data and convention.
Hardy-Weinberg equilibrium was confirmed in UKBB and H2000, while excess homozygosity (P<0.001) in FinnGen likely reflects population founder effects, not genotyping error.

The *SCN5A* variant T220I was directly genotyped within the UKBB and H2000, in FinnGen T220I was mostly directly genotyped with 20,272 individuals imputed (n-heterozygous=218, n-homozygous=12, INFO-score=0.988).

Supplementary Note 2 - Definition of QTc

In H2000, the association between QTc time remained significant after adjustment for other covariates (calcium level, TSH, diastolic blood pressure, systolic blood pressure, respiratory diseases, arrhythmia, other heart illnesses and first 4 PCs of ancestry), with an average 7.84 ± 2.55 ms decrease in QTc duration for variant carriers after adjustment (P=0.0021). Notably, we found systolic blood pressure ($P=2.03\times10^{-12}$) and unspecified heart disease (P=0.014), to influence QTc time. We found the results also robust to using an alternative QT correction method (-5.83 ± 2.64 ms, P=0.0027, Method: Framingham correction [36]).

Supplementary Note 3 - ECG Analysis

Automated ECG measurements (PR interval, P-wave duration, QRS duration, and QT interval) were available for 116,014 UKBB study participants, extracted either from 12-lead ECGs (n=67,620 with at least one measurement released centrally by UKBB) or 3-lead upright resting ECGs taken before a bicycle exercise test (n=60,755 with at least one measurement extracted using the GE Marquette 12SL ECG analysis program). We evaluated two sets of measurements: 1) measurements derived only from 12-lead ECGs and 2) measurements derived from either 12-lead or 3-lead ECGs, prioritizing 12-lead ECGs in participants who had measurements from both 12-lead and 3-lead ECGs (n=12,361). The H2000 cohort comprised 6,048 participants with ECG measurements [29] and included a health examination interview, a physical examination, and an extensive questionnaire. Collected samples include venous blood samples, measurements of height, weight, and blood pressure, psychometric tests, and a resting 12-lead ECG, amongst others. The clinical examination included the medical history, an assessment of drug prescriptions, the cardiovascular and pulmonary status. The ECG was automatically interpreted by the Social Insurance Institution's Research Center. Heart rate–corrected QT intervals were calculated using the Bazett formula [31], defined as $QTc=QT(ms)/\sqrt{RR(s)}$, to adjust the measured QT interval for the influence of heart rate variability.

Supplementary Note 4 - Sensitivity analyses medication usage

Our primary analysis used a Cox proportional hazards model to assess the association between the T220I variant and the risk of incident AFib. To test if this association was robust and independent of medication usage, we performed a sensitivity analysis by including baseline use of medications as covariates within the Cox model. We considered antiarrhythmics (ATC: C01BC04, C01BD01, C01BD07), beta-blockers (C07AB03, C07AB07, C07AG02, C07AB02, C07AA07), calcium channel blockers (C08DA01, C08DB01), and cardiac glycosides (C01AA05). After adjustment, the protective effect of T220I remained stable and significant (HR=0.59 ,95% CI 0.53 – 0.65, P<0.0003).

Supplementary Note 5 - Ethics Approvals

Uusimaa Ethics provided approval for the Health 2000 survey (No. 407/E3/2000). All survey participants provided written informed consent. Access to the Health 2000 survey dataset, and linked hospitalization and death records were granted by the Statistics Finland and National Institute for Health and Welfare (THL) in Finland.

Study subjects in FinnGen provided informed consent for biobank research, based on the Finnish Biobank Act. Alternatively, separate research cohorts, collected prior the Finnish Biobank Act came into effect (in September 2013) and start of FinnGen (August 2017), were collected based on study-specific consents and later transferred to the Finnish biobanks after approval by Fimea (Finnish Medicines Agency), the National Supervisory Authority for Welfare and Health. Recruitment protocols followed the biobank protocols approved by Fimea. The Coordinating Ethics Committee of the Hospital District of Helsinki and Uusimaa (HUS) statement number for the FinnGen study is Nr HUS/990/2017.

The FinnGen study is approved by Finnish Institute for Health and Welfare (permit numbers: THL/2031/6.02.00/2017, THL/1101/5.05.00/2017, THL/341/6.02.00/2018, THL/2222/6.02.00/2018, THL/283/6.02.00/2019, THL/1721/5.05.00/2019 and THL/1524/5.05.00/2020), Digital and population data service agency (permit numbers: VRK43431/2017-3, VRK/6909/2018-3, VRK/4415/2019-3), the Social Insurance Institution (permit numbers: KELA 58/522/2017, KELA 131/522/2018, KELA 70/522/2019, KELA 98/522/2019, KELA 134/522/2019, KELA 138/522/2019, KELA 2/522/2020, KELA 16/522/2020), Findata permit numbers THL/2364/14.02/2020, THL/4055/14.06.00/2020, THL/3433/14.06.00/2020, THL/4432/14.06/2020, THL/5189/14.06/2020, THL/5894/14.06.00/2020, THL/6619/14.06.00/2020, THL/209/14.06.00/2021, THL/688/14.06.00/2021, THL/1284/14.06.00/2021, THL/1965/14.06.00/2021, THL/5546/14.02.00/2020, THL/2658/14.06.00/2021, THL/4235/14.06.00/2021, Statistics Finland (permit numbers: TK-53-1041-17 and TK/143/07.03.00/2020 (earlier TK-53-90-20) TK/1735/07.03.00/2021, TK/3112/07.03.00/2021) and Finnish Registry for Kidney Diseases permission/extract from the meeting minutes on 4th July 2019.

The Biobank Access Decisions for FinnGen samples and data utilized in FinnGen Data Freeze 12 include: THL Biobank BB2017_55, BB2017_111, BB2018_19, BB_2018_34, BB_2018_67, BB2018_71, BB2019_7, BB2019_8, BB2019_26, BB2020_1, BB2021_65, Finnish Red Cross Blood Service Biobank 7.12.2017, Helsinki Biobank HUS/359/2017, HUS/248/2020, HUS/430/2021 §28, §29, HUS/150/2022 §12, §13, §14, §15, §16, §17, §18, §23, §58, §59, HUS/128/2023 §18, Auria Biobank AB17-5154 and amendment #1 (August 17 2020) and amendments BB_2021-0140, BB_2021-0156 (August 26 2021, Feb 2 2022), BB_2021-0169, BB_2021-0179, BB_2021-0161, AB20-5926 and amendment #1 (April 23 2020) and its modifications (Sep 22 2021), BB_2022-0262, BB_2022-0256, Biobank Borealis of Northern Finland_2017_1013, 2021_5010, 2021_5010 Amendment, 2021_5018, 2021_5018 Amendment, 2021_5015, 2021_5015 Amendment, 2021_5015 Amendment_2, 2021_5023, 2021_5023 Amendment, 2021_5023 Amendment_2, 2021_5017, 2021_5017 Amendment, 2022_6001, 2022_6001 Amendment, 2022_6006 Amendment, 2022_6006 Amendment, 2022_6006 Amendment_2, BB22-0067, 2022_0262, 2022_0262 Amendment, Biobank of Eastern Finland 1186/2018 and amendment 22§/2020, 53§/2021, 13§/2022, 14§/2022, 15§/2022, 27§/2022, 28§/2022, 29§/2022, 33§/2022, 35§/2022, 36§/2022, 37§/2022, 39§/2022, 7§/2023, 32§/2023, 33§/2023, 34§/2023, 35§/2023, 36§/2023, 37§/2023, 38§/2023, 39§/2023, 40§/2023, 41§/2023, Finnish Clinical Biobank Tampere MH0004 and amendments (21.02.2020 & 06.10.2020), BB2021-0140 8§/2021, 9§/2021, §9/2022, §10/2022, §12/2022, 13§/2022, §20/2022, §21/2022, §22/2022, §23/2022, 28§/2022, 29§/2022, 30§/2022, 31§/2022, 32§/2022, 38§/2022, 40§/2022, 42§/2022, 1§/2023, Central Finland Biobank 1-2017, BB_2021-0161, BB_2021-0169, BB_2021-0179, BB_2021-0170, BB_2022-0256, BB_2022-0262, BB22-0067, Decision allowing to continue data processing until 31st Aug 2024 for projects: BB_2021-0179, BB22-0067,BB_2022-0262, BB_2021-0170, BB_2021-0164, BB_2021-

0161, and BB_2021-0169, and Terveystalo Biobank STB 2018001 and amendment 25th Aug 2020, Finnish Hematological Registry and Clinical Biobank decision 18th June 2021, Arctic biobank P0844: AR

Supplementary Note 6 - Replication of T220I PheWAS results in the UK Biobank

In the UKBB (n=487,702), we replicated our PheWAS for T220I in ICD-10 subcategories I40.[0-9]–I50.[0-9]), where we found nominally significant effects in FinnGen. We identified nominally significant effects for paroxysmal AFib (OR=0.44, 95% CI: 0.20 – 0.99, P=0.046) and unspecified AFib (OR=0.6, 95% CI: 0.41 – 0.89, P=0.01). For broader ICD categories (I40 – I50) we found a protective effect of T220I on AFib (OR=0.6, 95% CI: 0.41–0.89, P=0.0008). While effects of T220I on sick sinus syndrome were not significant, effect sizes and direction of effect were similar to results in FinnGen (OR=1.88, 95% CI: 0.78 – 4.56, P=0.16).

Supplementary Note 7 - Post myocardial infarction mortality

We found that T220I increased all-cause mortality in the 2 years after a myocardial infarction event (see also Supplementary Figure S8), which is consistent with the effects of pharmacologic sodium channel blockade after myocardial infarction tested in the Cardiac Arrhythmia Suppression trial (CAST, Echt et al NEJM, 1991) [34]. In the CAST trial, mortality associated with class 1c sodium channel blocker usage was driven by cardiac arrhythmia related mortality. We however observed rather the opposite trend in our data as death due to cardiac arrhythmia was numerically lower (Heterozygotes HR=0.56 (95% CI: 0.23 – 1.36), P=0.200) when restricting to immediate, underlying or contributing causes of death together, but also when considering these categories separately. Considering other common causes of death after myocardial infarction, we did not see increased rates for mortality due to cardiovascular disease (Heterozygotes HR=1.02, 95% CI: 0.84 – 1.25, P=0.811), nor heart failure (Heterozygotes HR=0.67, 95% CI: 0.35 – 1.30, P=0.236).

Further exploring specific reasons for the excess mortality, we compared causes of deaths of different causal categories (underlying, immediate and contributing) after MI associated with T220I. We found no significant effects of T220I on death due to AFib (minimum P=0.09), heart failure (minimum P=0.2) or other cardiovascular disease (when excluding heart failure and AFib). While this was not statistically significant, we found an unusually high number of contributing and immediate causes of death of other diseases such as chronic obstructive pulmonary disease or other pneumological diseases that may drive the effect on all-cause mortality after MI. We thus cannot further explain the increased mortality associated with T220I after MI. As a limitation of our data, the increased mortality could have been driven by other causes of death that were insufficiently captured with the data available to us from the Finnish death registries (provided by Statistics Finland). Registry data may also not be sensitive for capturing all deaths from cardiac arrhythmias.

Supplementary Figures

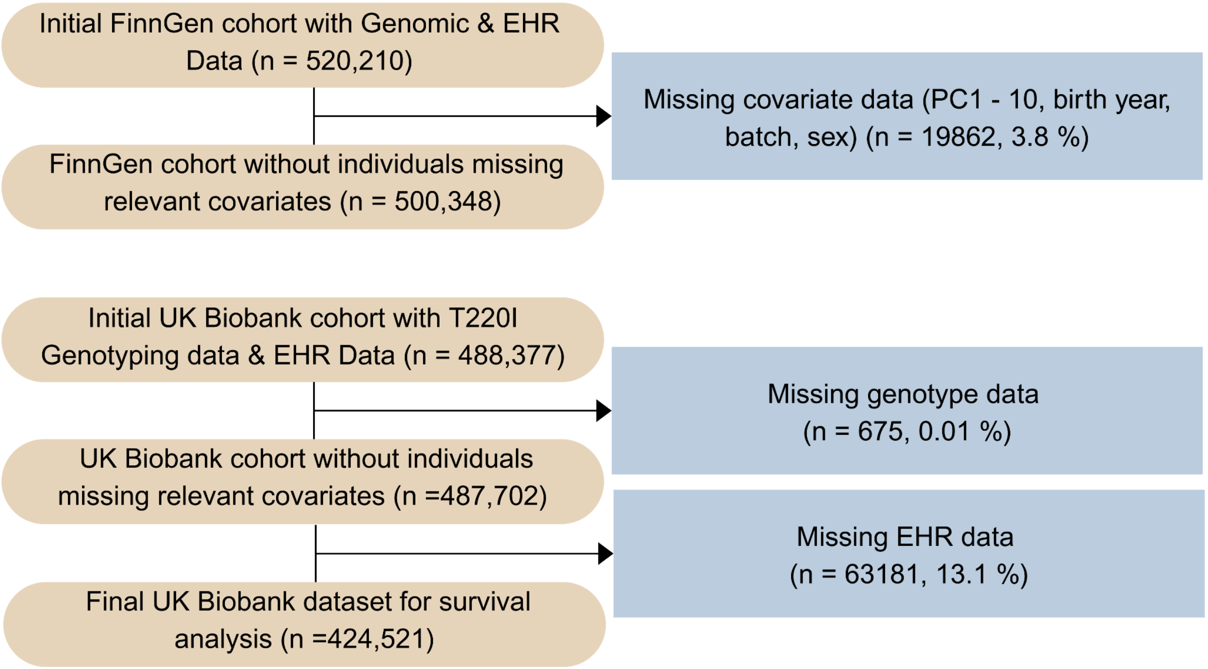

Supplementary Figure S1 Flowchart of exclusion criteria for the FinnGen cohort and the UK Biobank, survival analysis. From an initial population of 520,210 individuals in FinnGen (DF 12) and 488,377 in the UK Biobank (DF September 2022) with available genomic and EHR data, individuals were excluded based on missing genotype and covariate data, resulting in a final cohort of 500,348 individuals in FinnGen and 424,521 in the UK Biobank.

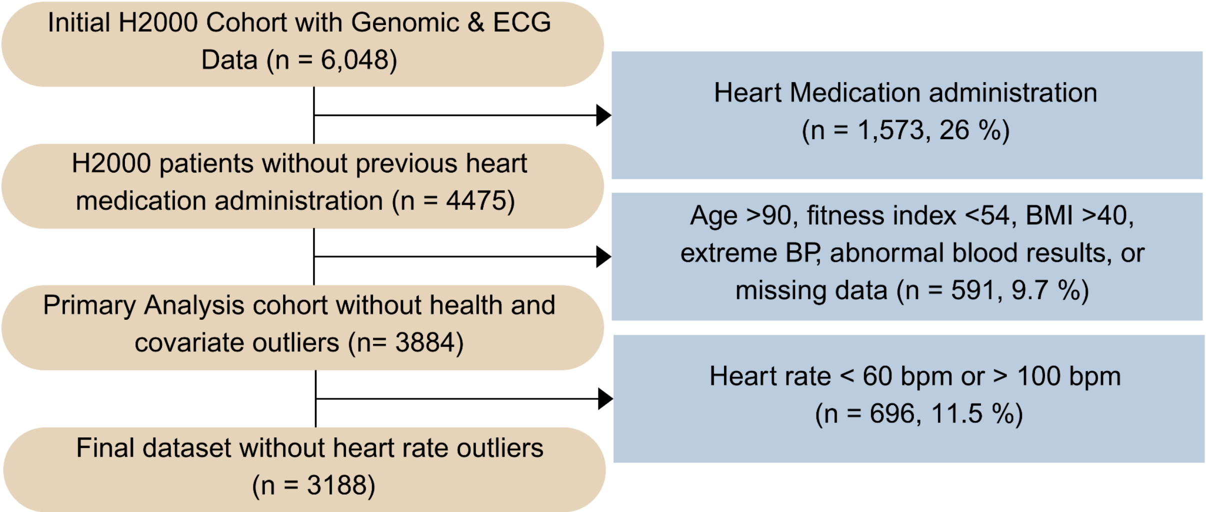

Supplementary Figure S2 Flowchart of exclusion criteria for the H2000 cohort, ECG analysis. From an initial population of 6,048 individuals with available genomic and electrocardiogram (ECG) data, individuals were excluded based on heart medication administration (n=1,573), predefined health and covariate outliers (n=591), and heart rate outliers (n=696), resulting in a final cohort of 3,188 individuals.

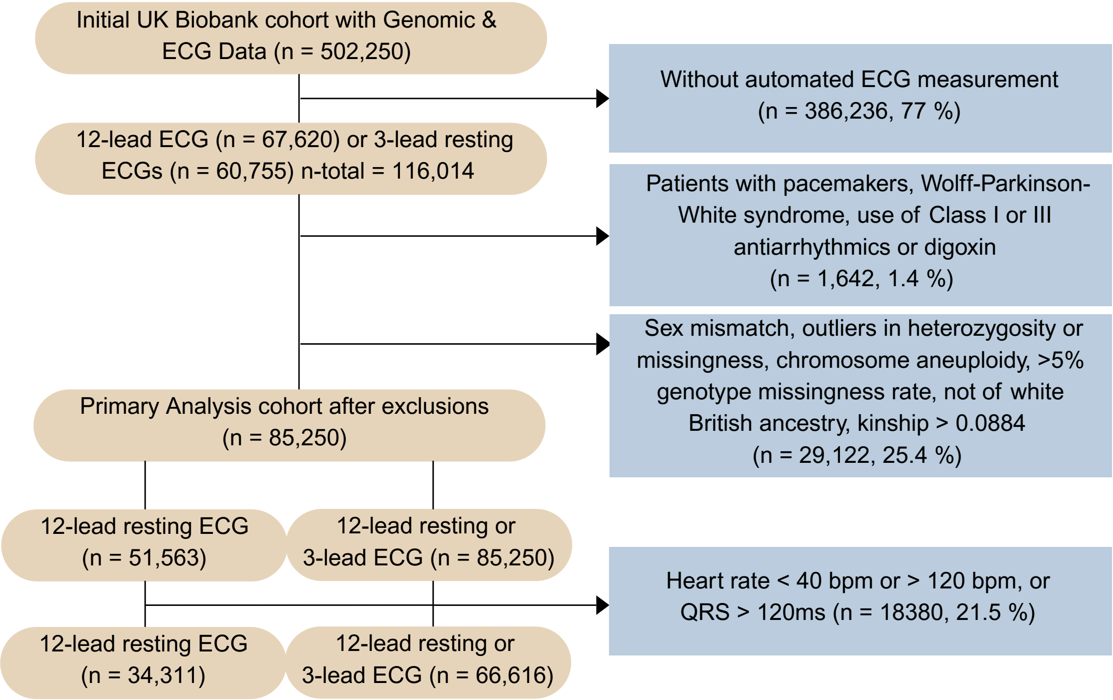

Supplementary Figure S3 Flowchart of exclusion criteria for the UK Biobank cohort, ECG analysis. From an initial population of 502,250 individuals, we selected individuals with available ECG measurements, with available genomic and electrocardiogram (ECG) data (n=116,014), individuals were excluded based on heart medication administration (n=1,573), predefined health and covariate outliers (n=591), and heart rate outliers (n=696), resulting in a cohort of 82,250 individuals. Resting heart rate outliers were removed resulting in a final cohort of 66,616.

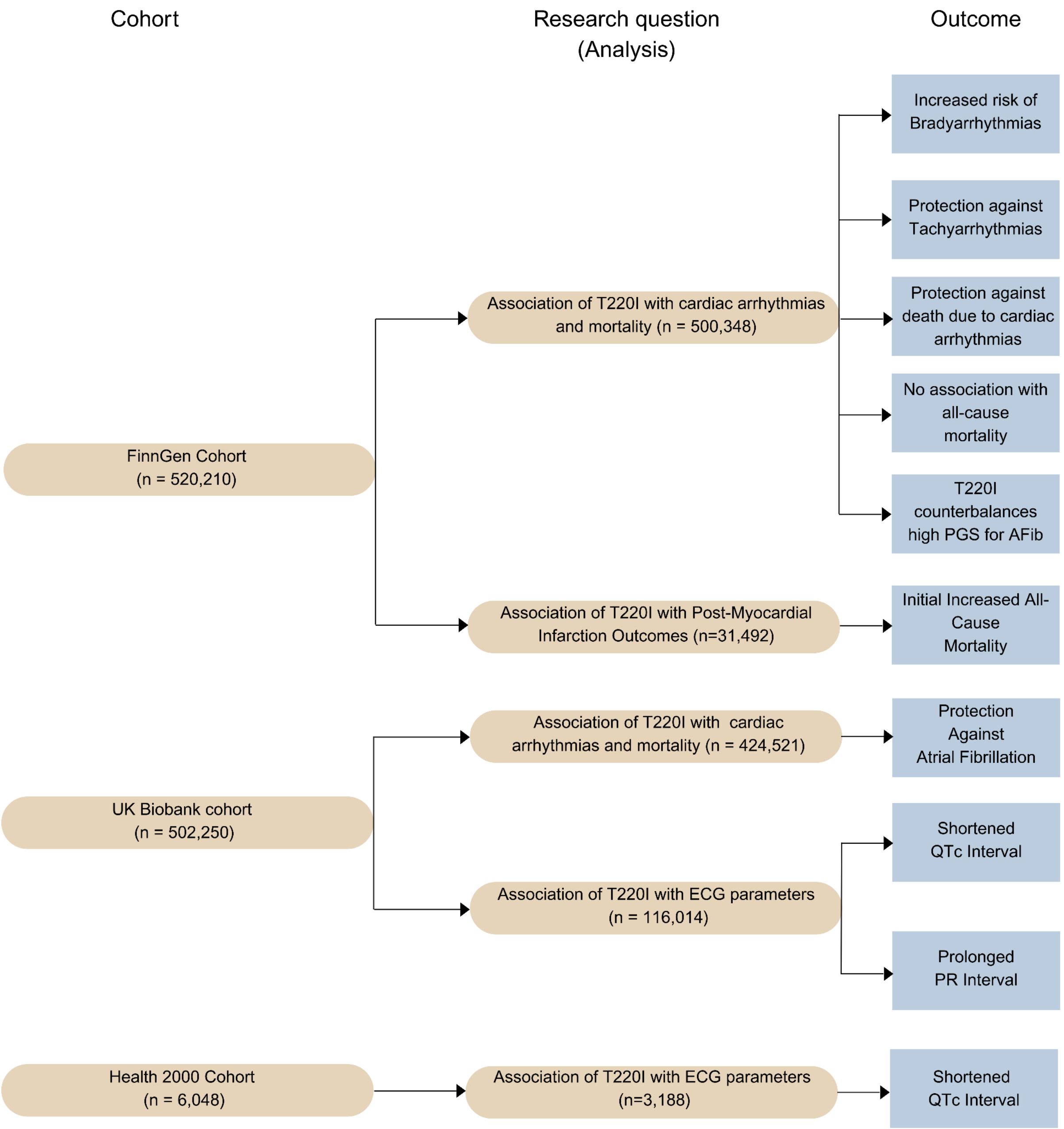

Supplementary Figure S4 Flowchart of the multi-cohort study design. We utilized three large-scale biobanks to assess the effects of the T220I variant on different phenotypes and ECG parameters. Primary discovery for disease associations, survival, and post-myocardial infarction outcomes was conducted in the FinnGen cohort (n=520,210), cardiac disease associations were replicated in the UK Biobank (n=502,250), which was also used for detailed ECG analysis. Further ECG analyses were performed in the Health 2000 (H2000) cohort (n=6,048).

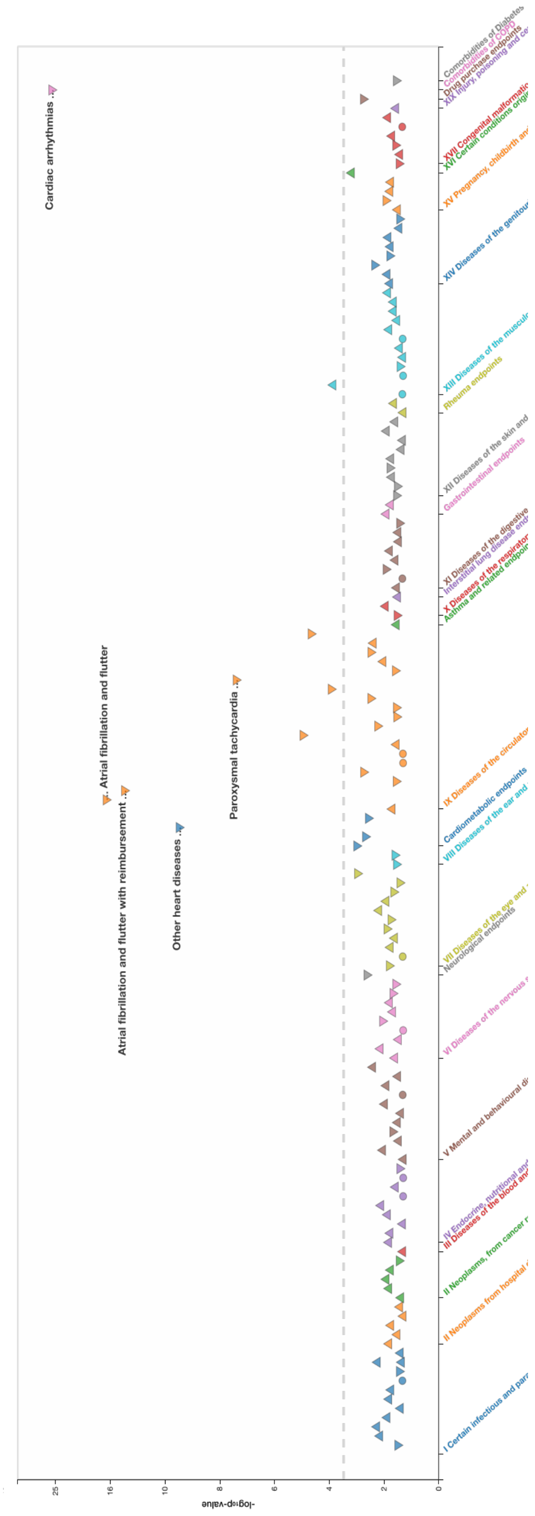

Supplementary Figure S5. Phenome-wide-association study of T220I. -log10 p-values (y-axis) of associations of the *SCN5A* variant T220I with all available FinnGen phenotypes (n=2,469, grouped by broader FinnGen phenotype categories). The grey dashed line shows the multiple testing corrected statistical significance threshold (P=3×10^{-4}). Colors denote different disease categories as specified on the x-axis. Triangles pointing upwards indicate a risk-increasing association while triangles pointing downwards indicate a negative association.

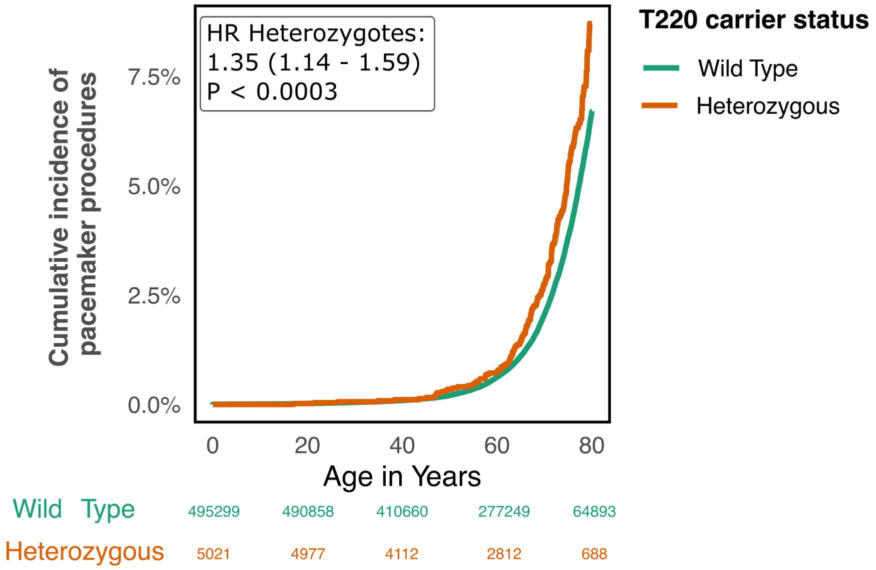

Supplementary Figure S6 Cumulative incidence of first pacemaker procedure, stratified by carrier state of T220I. Cumulative disease incidence is shown on the y-axis. Hazard ratio with 95% CIs and P-value is shown inside the plot.

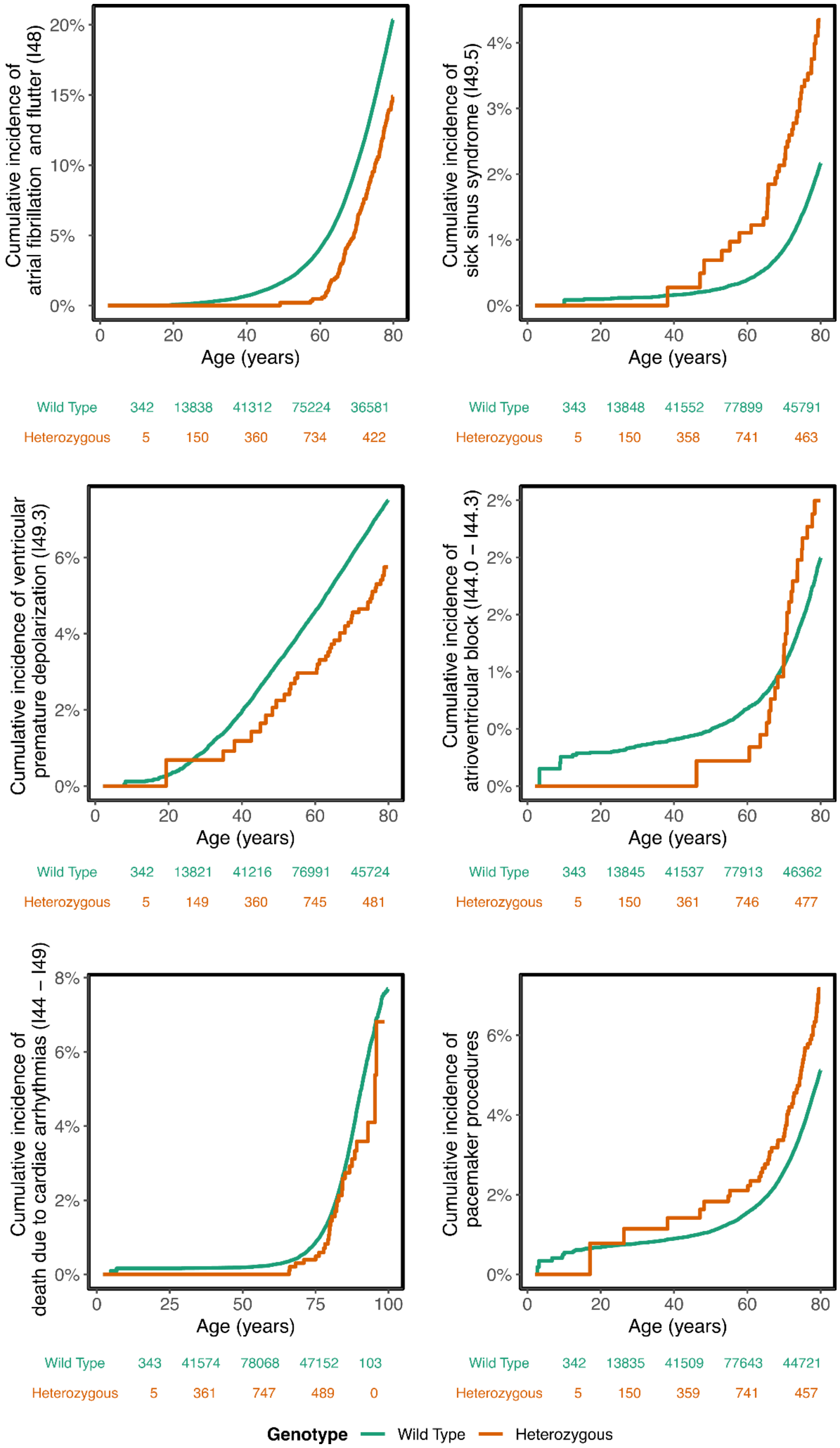

Supplementary Figure S7. Cumulative incidence of cardiac phenotypes, stratified by carrier states of T220I and using competing risk analysis adjusting for other causes of death and adjusting for left truncation. Panels show the cumulative incidence of the respective event (y-axis) across the lifetime (age in years, x-axis) in T220I heterozygotes (orange) and wild types (green), respectively.

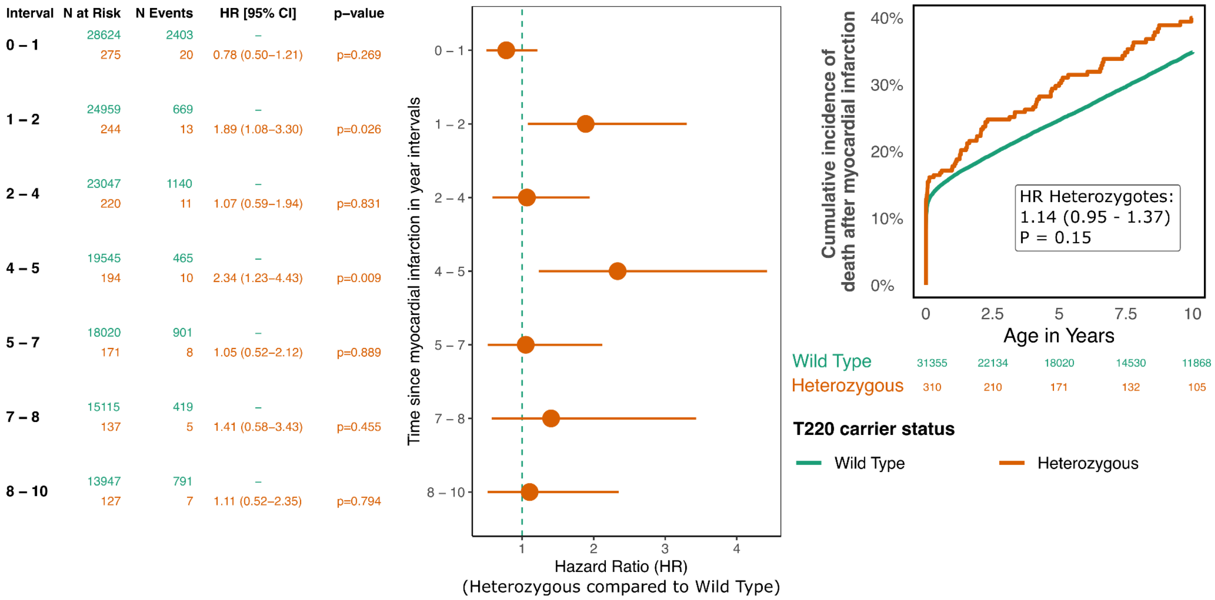

Supplementary Figure S8 T220I increased all-cause mortality after myocardial infarction. Left panel shows hazard ratios of all-cause mortality post myocardial infarction for T220I carriers, measured in intervals of one year, stratified by carrier states of T220I. Right panel shows cumulative incidence of death (y-axis) in the time (x-axis, years) after a myocardial infarction event heterozygotes showed an increased risk for all-cause mortality, with stronger effects early after a myocardial infarction.

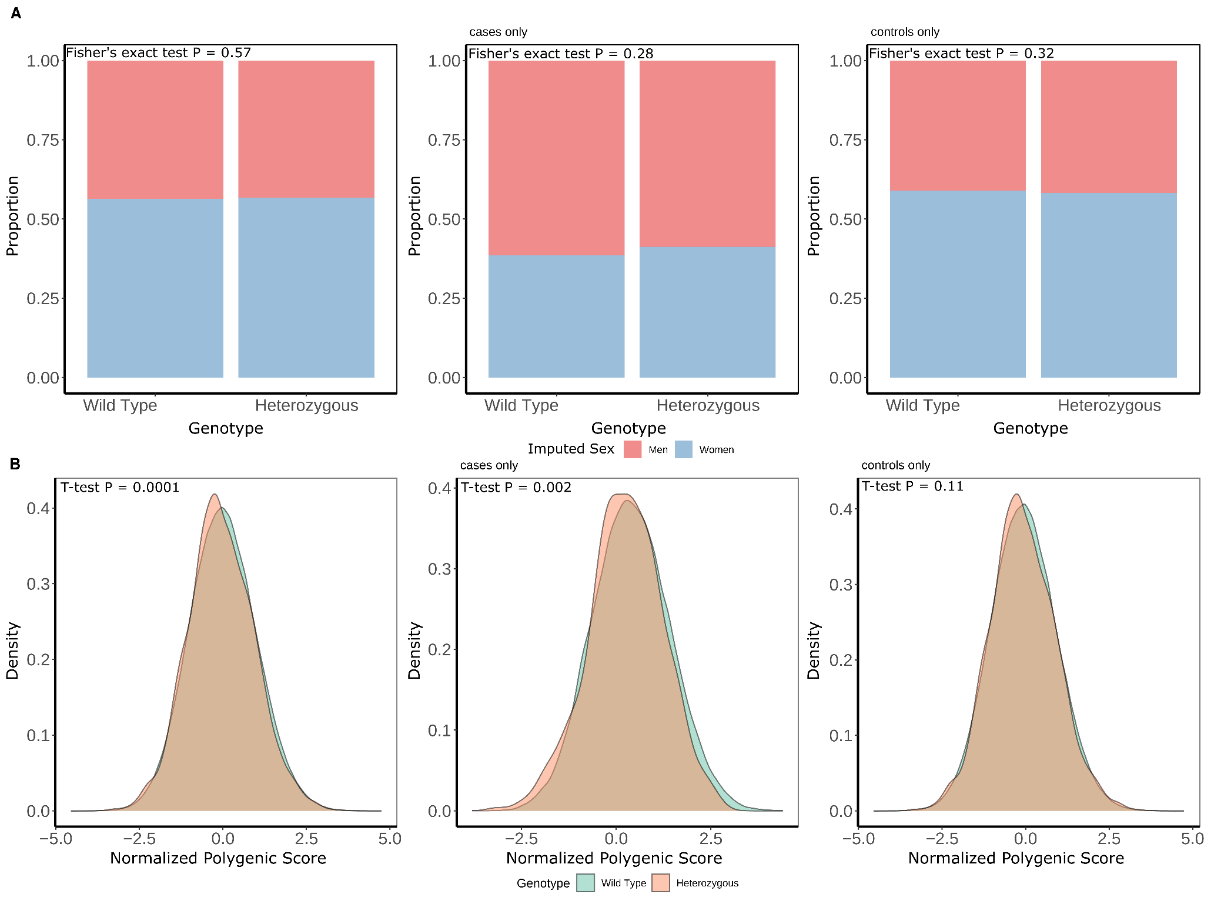

Supplementary Figure S9. Sex and AFib PGS distributions for T220I Heterozygotes and Wild types in FinnGen DF12. (A) Proportion of Females and Males for all individuals, cases of AFib and controls of AFib. With Fisher's exact test, there were no significant differences for the distribution of sexes between heterozygotes and wild types (P=0.569, n=500,348). (B) Density plots of an AFib PGS for all individuals, cases of AFib and controls of AFib. There were significant differences in the distribution of an AFib PGS between heterozygotes and wild types in all individuals (T-test, P<0.001, n=500,348) and cases only (P=0.002).

Supplementary Tables

Supplementary Table S1 Summary of studies investigating the *SCN5A* T220I variant and its association with cardiac arrhythmias Abbreviations: AFib, atrial fibrillation; DCM: Dilated cardiomyopathy; SSS: sick sinus syndrome.

Study	Sample Size	Study Design	Main Findings	Cohorts/Database Used
Heyne et al., 2023 [13]	~ 190,000 (FinnGen) ~ 500,000 (UK Biobank)	Population-based, longitudinal	Protective effect against AFib (P<0.001); increased risk for SSS in homozygotes (P<0.001);	FinnGen, UK Biobank
Benson et al., 2003 [19]	One family (n=2 affected variant carriers)	Family study	Linked compound heterozygous *SCN5A* mutations, including T220I, to congenital (early-onset) SSS in children.	Individual, family-based genetic data + electrophysiology experiments
Gui et al., 2010 [20]	-	Functional study	Demonstrated altered channel kinetics contributing to SSS; identified multiple LoF mechanisms in *SCN5A* mutations, including T220I.	Electrophysiology experiments
Olson et al., 2005 [11]	One family (n=2 variant carriers with DCM, 2 healthy variant carriers with 'larger hearts')	Family study	Association between T220I and DCM; conduction delay of T220I	Selected (DCM) patient populations
Olesen et al., 2012 [24]	Case-report (n=1)	Observational, ECG-focused	Association between T220I and early-onset lone AFib; mild LoF effects on $Na_V1.5$ confirmed.	Selected (AFib) patient populations
Baskar et al., 2014 [18]	Case report (n=1)	Family study	Reported an 11-year-old girl with atrial standstill carrying compound heterozygous *SCN5A* mutations, including T220I.	Individual family-based genetic data

Supplementary Table S2 ICD-10 diagnostic codes used to define cardiac disease endpoints.

Supplementary Table S3 Summary of covariates for the H2000 study after exclusions.

Characteristic	Wild-type (n=3,159)	Heterozygous (n=29)
Sex		
- Male	n=1,416	n=17
- Female	n=1,743	n=12
Age, years	47.7 (12.3)	47.4 (10.4)
Calcium, mmol/L	2.4 (0.1)	2.4 (0.1)
Thyrotropin, mU/L	1.5 (0.8)	1.7 (0.8)
BMI, kg/m²	26.2 (4.2)	26.8 (3.2)
Fitness index score	83.8 (4.9)	84.9 (5.2)
Heart rate (bpm)	70.5 (8.3)	69.7 (7.3)

Supplementary Table S4 Associations between significantly associated phenotypes in FinnGen. Abbreviations: OR, odds-ratio; CI, Confidence interval.

Name	OR (95% CI)	P
Dilated cardiomyopathy (I42.0)	0.66 (0.46 – 0.95)	0.026
Atrioventricular block, first degree (I44.0)	1.48 (1.03 – 2.13)	0.035
Atrioventricular block, complete (I44.2) (Heterozygous)	1.32 (1.01 – 1.74)	0.042
Atrioventricular block, complete (I44.2) (Homozygous)	1.83 (0.66 – 5.08)	0.248
Left bundle-branch block, unspecified (I44.7)	0.5 (0.29 – 0.87)	0.013
Bifascicular block (I45.2)	7.89 (3.63 – 17.12)	<0.001
Conduction disorder, unspecified (I45.9)	3.45 (1.25 – 9.52)	0.017
Supraventricular tachycardia (I47.1)	0.53 (0.40 – 0.71)	<0.001
Ventricular tachycardia (I47.2)	0.45 (0.28 – 0.74)	0.002
Atrial fibrillation and flutter (I48)	0.57 (0.51 – 0.64)	<0.001
Paroxysmal atrial fibrillation (I48.0)	0.52 (0.41 – 0.66)	<0.001
Persistent atrial fibrillation (I48.1)	0.61 (0.43 – 0.88)	0.009
Chronic atrial fibrillation (I48.2)	0.60 (0.41 – 0.88)	0.009
Atrial fibrillation and atrial flutter, unspecified (I48.9)	0.48 (0.37 – 0.63)	<0.001
Atrial premature depolarization (I49.1)	0.67 (0.49 – 0.90)	0.008
Ventricular premature depolarization (I49.3)	0.51 (0.41 – 0.64)	<0.001
Other or unspecified premature depolarization (I49.4)	0.64 (0.47 – 0.85)	0.003
Sick sinus syndrome (I49.5) (Heterozygous)	2.36 (1.98 – 2.81)	<0.001
Sick sinus syndrome (I49.5) (Homozygous)	3.81 (2.22 – 6.56)	<0.001
Cardiac arrhythmia, unspecified (I49.9)	0.72 (0.62 – 0.82)	<0.001
Heart failure (I50)	0.69 (0.48 – 0.97)	0.034
Left ventricular failure (I50.1)	0.75 (0.59 – 0.96)	0.020
Heart failure, unspecified (I50.9)	0.84 (0.74 – 0.96)	0.012

Supplementary Table S5 Associations between electrophysiological parameters and T220I across different ECG setups in the UK Biobank. Abbreviations: QTc, Bazett–corrected QT interval; QRS, QRS complex duration; PR, PR interval; Pdur, P-wave duration.

Measurement	Data source	Beta [ms]	Lower CI [ms]	Upper CI [ms]	P	N total
QTc	12-lead resting ECG	-4.20	-9.68	1.29	0.13	35565
QTc	12-lead resting ECG before exercise test Excluding QRS>120 ms, HR<40, or HR>120	-4.95	-10.29	0.39	0.07	34140
QRS	12-lead resting ECG	-1.48	-4.02	1.05	0.25	51563
PR	12-lead resting ECG	11.98	5.86	18.10	0.0001	33674
Pdur	12-lead resting ECG	2.63	-0.47	5.73	0.097	48805
QTc	12-lead resting or 3-lead upright ECG before exercise test	-3.25	-7.13	0.64	0.1	71402
QTc	12-lead resting or 3-lead upright ECG before exercise test Excluding QRS>120 ms, HR<40, or HR>120	-4.41	-8.22	-0.60	0.02	66616
QRS	12-lead resting or 3-lead upright ECG before exercise test	-0.07	-3.71	3.57	0.97	85250
PR	12-lead resting or 3-lead upright ECG before exercise test	6.15	1.98	10.31	0.0038	69605
Pdur	12-lead resting or 3-lead upright ECG before exercise test	1.24	-1.1	3.59	0.3	82688
